# Distribution in Different Organisms of Amino Acid Oxidases with FAD or a Quinone As Cofactor and Their Role as Antimicrobial Proteins in Marine Bacteria

**DOI:** 10.3390/md13127073

**Published:** 2015-12-16

**Authors:** Jonatan C. Campillo-Brocal, Patricia Lucas-Elío, Antonio Sanchez-Amat

**Affiliations:** Department of Genetics and Microbiology, Faculty of Biology, University of Murcia, Murcia 30100, Spain; jonatancristian.campillo@um.es (J.C.C.-B.); patlucel@um.es (P.L.-E.)

**Keywords:** amino acid oxidases, quinone cofactor, flavoprotein, antimicrobial

## Abstract

Amino acid oxidases (AAOs) catalyze the oxidative deamination of amino acids releasing ammonium and hydrogen peroxide. Several kinds of these enzymes have been reported. Depending on the amino acid isomer used as a substrate, it is possible to differentiate between l-amino acid oxidases and d-amino acid oxidases. Both use FAD as cofactor and oxidize the amino acid in the alpha position releasing the corresponding keto acid. Recently, a novel class of AAOs has been described that does not contain FAD as cofactor, but a quinone generated by post-translational modification of residues in the same protein. These proteins are named as LodA-like proteins, after the first member of this group described, LodA, a lysine epsilon oxidase synthesized by the marine bacterium *Marinomonas mediterranea*. In this review, a phylogenetic analysis of all the enzymes described with AAO activity has been performed. It is shown that it is possible to recognize different groups of these enzymes and those containing the quinone cofactor are clearly differentiated. In marine bacteria, particularly in the genus *Pseudoalteromonas*, most of the proteins described as antimicrobial because of their capacity to generate hydrogen peroxide belong to the group of LodA-like proteins.

## 1. Introduction

**Figure 1:**
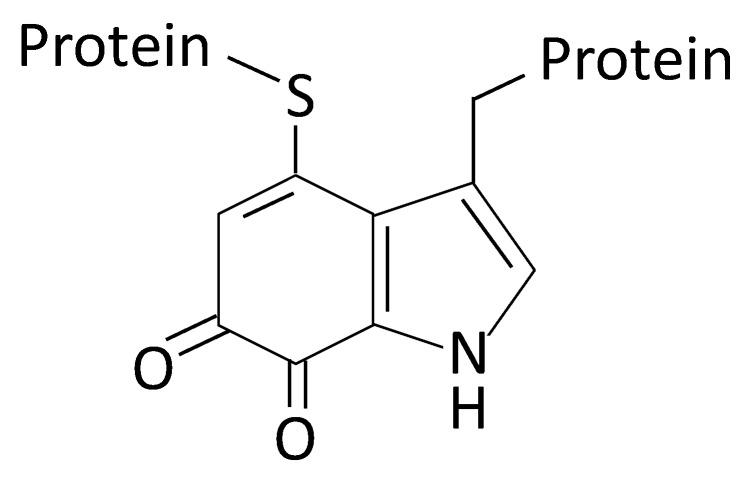
Cysteine tryptophylquinone (CTQ) cofactor.

In a broad sense, amino acid oxidases (AAOs) can be described as enzymes that oxidize amino acids releasing ammonium and hydrogen peroxide. Two big groups of these enzymes are recognized depending on the chirality of the amino acid used as substrate. l-Amino acid oxidases, EC 1.4.3.2, (commonly abbreviated as LAAOs or LAOs) are flavoenzymes that oxidize l-amino acids releasing the corresponding α-keto acid in addition to ammonium and hydrogen peroxide. LAAOs are distributed in many biological groups. The best characterized members of this family have been studied in snake venoms [1]. Additionally, several enzymes with this activity have also been described in many other groups including bacteria [2]. d-Amino acid oxidases (DAAOs or DAOs), EC 1.4.3.3, are flavoproteins showing strict specificity for d-amino acids [3,4]. These enzymes are also broadly distributed in different groups of organisms with distinct physiological roles. Glycine is the unique amino acid that has no enantiomers. In *Bacillus*, a glycine oxidase has been described [5]. Recently, a novel class of AAOs has been reported. The marine bacterium *Marinomonas mediterranea* synthesizes LodA, a l-lysine epsilon-oxidase (EC 1.4.3.20) [6,7]. The cofactor of LodA is cysteine tryptophylquinone (CTQ) (Figure 1), a type of quinone cofactor generated by the post-translational modification of two residues in the same protein [8,9]. GoxA is an enzyme with sequence similarity to LodA and the same kind of cofactor, but it shows glycine oxidase activity [10,11]. The analysis of sequenced microbial genomes has revealed that, approximately, 1% of them contain genes encoding proteins similar to LodA and GoxA [12].

Enzymes with AAO activity are of great scientific interest because of the number of physiological activities displayed in different organisms. For example, the generation of hydrogen peroxide has been described from a physiological point of view as involved in antimicrobial processes. Among these processes, bacterial biofilm development [13], microbial competition related to biocontrol processes in fungi [14], protection of fish skin bacterial infections [15] and participation in the human immune system [16] are included. AAOs are also of interest in relation to their biotechnological applications [4,17]. Recently, their potential interest in medicine as antitumorals and as antimicrobials has increased [18,19]. In both cases, the hydrogen peroxide generated as product of the reaction plays a very important role.

LAAOs have been known for more than half a century. In many cases, when an enzyme showed that activity it was assumed to be a flavoprotein. However, it is important to point out that not in all the cases it has been demonstrated that the activity is due to an enzyme with a flavin cofactor. It is also important to bear in mind that the enzymatic activity of a protein may depend on the presence of other compounds, as it has been described for an enzyme whose activity can change from l-amino acid oxidase to monooxygenase by either mutation or change in the conditions of the assay [20]. Besides, it is well known that many enzymes may show latent promiscuous activities, and that the actual enzymatic activity could be different to the one initially described. As an example, an enzyme from *Streptococcus oligofermentans* was initially described as an l-amino acid oxidase [21] and lately reclassified as an aminoacetone oxidase involved in antioxidant mechanisms [22].

The aim of this review has been to study the phylogenetic relationship of all the enzymes described as amino acid oxidases, including the new family with quinone cofactor, in order to facilitate future work on novel enzymes with that activity. In the last section, the enzymes synthesized by marine microorganisms described as antimicrobial proteins will be discussed.

## 2. Phylogenetic Analysis of Proteins with Amino Acid Oxidase Activity

**Figure 2:**
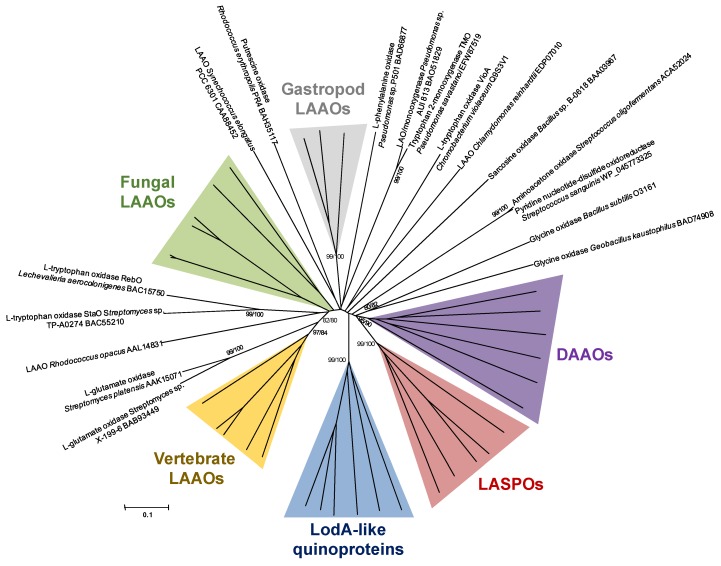
Phylogenetic relationships of enzymes with amino acid oxidase activity. The tree was created by the Neighbor-Joining method integrated in the program MEGA6 [23]. Sequences were aligned using the program MUSCLE built in MEGA6. The evolutionary distances were computed using the *p*-distance method and are in the units of the number of amino acid differences per site. Numbers at branches indicate bootstrap values higher than 70% for both Neighbor-Joining and Maximum Likelihood trees. The colored groups are detailed in Figure 3 and Figure 4. LAAOs, l-amino acid oxidases; DAAO, d-amino acid oxidases; LASPOs, l-aspartate oxidases.

A bibliographic search has been performed in order to obtain representative sequences of proteins with amino acid oxidase activity. All the microbial proteins considered are shown in supplementary Table 1. Table S1 includes proteins characterized at the molecular level, as well as other enzymes in which only the enzymatic activity has been reported. In order to shed light on the evolutionary relationships of AAOs, a phylogenetic analysis has been performed that includes the microbial proteins for which the encoding gene has been cloned, plus other representative proteins from higher organisms (Figure 2). Those AAOs from higher organisms are listed in supplementary Table 2.

It has been possible to recognize several closely related groups which are named after their common characteristic. The proteins similar to LodA containing a quinone cofactor form a phylogenetic group clearly differentiated from all the other enzymes. Among the rest of enzymes with AAO activity, DAAOs from different organisms constitute a well-defined cluster. Another group clearly differentiated is the one containing l-aspartate oxidases. Regarding LAAOs, they constitute a broad group distributed in different organisms where they have evolved to meet different physiological functions. In the next sections, the different groups will be discussed separately.

### 2.1. AAOs with a Quinone Cofactor (LodA-Like Proteins)

l-Lysine ε-oxidase (LodA) synthesized by the melanogenic marine bacterium *M. mediterranea* was the first quinoprotein reported with LAAO activity [7]. LodA received a new number by the Enzyme Commission (EC 1.4.3.20) since it catalyzes the oxidative deamination of the amine group in epsilon position of l-Lys [6]. It has been demonstrated that the cofactor of LodA is cysteine tryptophylquinone (CTQ) (Figure 1), which is generated by post-translational modification of residues in the same protein [9,11]. In the post-translational modification of LodA, the flavoprotein LodB, encoded in the same operon, plays an important role [8,24]. Regarding its physiological function, it has been shown that LodA plays a role, mediated by the hydrogen peroxide generated, in the development and differentiation of microbial biofilms [13].

Another quinoprotein with AAO activity was identified in *M. mediterranea*. This is a novel glycine oxidase (GoxA) which shows important differences in terms of substrate range and sensitivity to inhibitors to other glycine oxidases previously described [10]. GoxA is more specific for glycine and structurally different since it also contains the cofactor CTQ [11]. Genome mining revealed genes encoding proteins similar to LodA/GoxA in a number of microbial genomes. They are mostly present in *Bacteria*, absent in *Archaea* and, in *Eukarya*, only detected in a small group of fungi of the class *Agaromycetes* [12]. This new family of proteins has been named as the LodA-like family of proteins. Sequence alignment of the LodA-like proteins allowed the detection of several conserved residues. All these proteins showed a Cys and a Trp that aligned with the residues that are forming part of the CTQ cofactor in LodA and GoxA. Thus, this observation strongly suggests that proteins of the LodA-like family may contain a CTQ quinone cofactor [12]. Apart from LodA and GoxA, various oxidases of this group have been characterized in marine bacteria as discussed later (Section 3).

### 2.2. d-Amino Acid Oxidases

**Figure 3:**
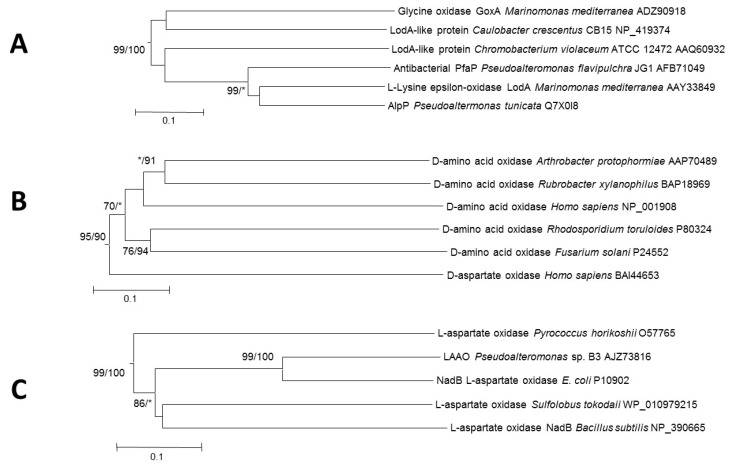
Phylogenetic relationships of representative LodA-like proteins (**A**), d-amino acid oxidases (**B**) and l-aspartate oxidases (**C**); The tree was created by the Neighbor-Joining method integrated in the program MEGA6 [23]. Sequences were aligned using the program MUSCLE built in MEGA6. The evolutionary distances were computed using the *p*-distance method and are in the units of the number of amino acid differences per site. Numbers at branches indicate bootstrap values higher than 70% for both Neighbor-Joining and Maximum Likelihood trees. An asterisk indicates that this branch was not detected, or it had a value lower than 70%.

d-Amino acid oxidases (EC 1.4.3.3) are FAD-containing proteins with a strict stereospecificity. They catalyze the oxidative deamination of neutral and basic d-amino acids to give α-keto acids, ammonium and hydrogen peroxide [3,25]. However, DAAOs show a negligible or no activity towards acidic d-amino acids, which are substrates for the closely related animal flavoproteins d-aspartate oxidases (DDO, also abbreviated as DASPO; EC 1.4.3.1) [26] (Figure 3B). DAAOs also cluster close to the glycine oxidases group (Figure 2). DAAO activity has been described in a wide variety of organisms such as bacteria [27], fungi [28], plants [29], nematodes [30], fishes [31], and mammals, including humans [32]. DAAOs participate in different physiological functions [33]. In yeast, they have a catabolic role since DAAOs allow yeast cells to grow on d-amino acids. In humans, DAAOs manage the levels of the neuromodulator d-serine of NMDA (*N*-methyl-d-aspartate) receptors, which are related with many pathological processes. However, the physiological role of bacterial DAAOs is still largely unknown [25]. DAAOs can be utilized for a broad range of applications, such as the determination of d-amino acids, production of building blocks for pharmaceuticals, synthesis of α-keto acids, and diagnosis and treatment of certain diseases [4,34].

### 2.3. l-Aspartate Oxidases

l-Aspartate oxidases (LASPOs; EC 1.4.3.16) are flavoproteins encoded by the gene *nadB* that play an important role in NAD^+^ synthesis in the cells, as they catalyze the formation of iminoaspartate from l-aspartate, the first step in the *de novo* biosynthesis of NAD^+^ in many bacterial, archaeal and plant cells [35]. NadB forms a reversible multienzyme complex with NadA, which carries on the transformation leading to quinolinic acid, an intermediary in the NAD^+^ synthetic pathway. The high activity of NadB seems to imply an oxidative stress for the cells, due to the generation of hydrogen peroxide [36]. It is noteworthy that, under physiological conditions, LASPOs do not produce ammonia, since the final product of the pathway conserves the original *N*-atom of l-aspartic acid. In this feature, they do not conform to the classical definition of amino acid oxidases. Interestingly, NadB function is substituted by an NADP-dependent l-aspartate dehydrogenase in *Thermotoga maritima* which has no sequence similarities with any amino acid oxidase. Moreover, the gene encoding that enzyme is next to *nadA* in the genome, as described in *Bacillus* [37].

Sequence analysis reveals that LASPOs have some similarities with fumarate reductases and succinate dehydrogenases in terms of folding topology and conformation of the FAD-binding site and active center, suggesting a similar catalytic mechanism [38,39] and a common evolutionary origin [40]. This implies that l-aspartate oxidases are different from other flavin-dependent amino acid oxidases, such as the d-amino acid oxidases. This fact is reflected as a separate and defined cluster in the phylogenetic tree (Figure 2 and Figure 3C).

One of the similarities between LASPOs and the succinate dehydrogenase/fumarate reductase oxidoreductase family is their ability to use different electron acceptors [41]. Accordingly, NadB can use either molecular oxygen or fumarate as electron acceptors of the reaction, permitting aspartate oxidation in anaerobic conditions [42]. Other aspartate oxidase, such as those from archaea, are being widely proposed for bioanalytical methods due to their high thermostability [43,44]. For instance, the LASPO from *Sulfolobus tokodaii*, which has been recombinantly expressed, has a broad pH range of activity and shows a weak inhibition pattern. This enzyme not only has a high thermal stability but also binds tightly the FAD cofactor [43].

Although aspartate oxidases were proposed as potential drug targets, since they are absent in mammals [38], this research has not been followed, probably because prokaryotes may have alternative routes for NAD^+^ production [45]. On the other hand, the gene is considered as an antivirulence loci (AVL), either inactivated or lost in all pathogenic *Shigella* strains studied, as the presence of quinolinic acid is detrimental to its invasive process [46]. This pathoadaptive evolution is accompanied by a nicotinic acid auxotrophy that bypasses the need of quinolinic acid production in the NAD^+^ synthetic pathway.

### 2.4. LAAOs in Animals

The LAAOs from animals clusters in two different groups. One of them includes enzymes from vertebrates, and the other group includes gastropod enzymes (Figure 2). This observation is in agreement with previous studies that indicated that those enzymes may have evolved separately in the innate immune system of both groups [18,47].

#### 2.4.1. LAAOs from Vertebrates

**Figure 4:**
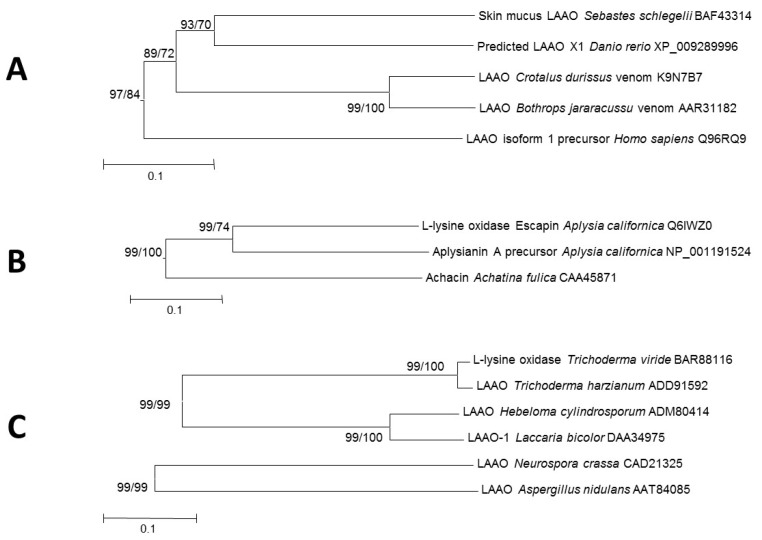
Phylogenetic relationships of representative l-amino acid oxidases in vertebrates (**A**), gastropods (**B**) and fungi (**C**). The tree was created by the Neighbor-Joining method integrated in the program MEGA6 [23]. Sequences were aligned using the program MUSCLE built in MEGA6. The evolutionary distances were computed using the *p*-distance method and are in the units of the number of amino acid differences per site. Numbers at branches indicate bootstrap values higher than 70% for both Neighbor-Joining and Maximum Likelihood trees.

The phylogenetic analysis of AAOs revealed a cluster including enzymes from snake venoms, fishes and mammals (Figure 2 and Figure 4A). Venom LAAOs have been subject of study for many years and several reviews have been published dealing with their biochemical properties and their interest in pharmacology [1,48]. Venom LAAOs show preference towards hydrophobic amino acids such as l-Leu, l-Phe and l-Met [48]. Numerous biological effects, such as antiparasitic, antimicrobial, apoptotic, *etc.*, have been reported for those enzymes [1,49].

The skin of the fish is a barrier preventing the infection by microorganisms. The mucus layer contains many peptides and proteins that play a role in that process. In this layer, LAAOs specific for l-lysine have been detected in different fishes such as the rockfish *Sebastes schlegelii* [15] and the great sculpin *Myoxocephalus polyacanthocephalus* [50]. The *Sebastes* LAAO has been used for the design of a biosensor for l-Lys [51]. LAAOs are not only present in the skin mucus but also in other tissues and the serum of fishes [52,53]. The induction of the LAAO of the Atlantic cod *Gadus morhua* after exposure of the fish to pathogenic bacteria supports its involvement in antibacterial defense [54].

Regarding mammals, the interleukin 4-induced gene (IL4I1) codes for an l-amino acid oxidase with strong preference for l-phenylalanine as substrate, which seems to act as a regulator of the immune system [55]. IL4I1 shows antimicrobial properties that have been associated to the hydrogen peroxide generated, the basification of the medium due to ammonia accumulation and the deprivation of the amino acid [16]. A LAAO has been detected in the milk of the mouse with substrate similarity to the human enzyme that has been proposed to participate in the protection against bacterial infections [56].

#### 2.4.2. LAAOs in Gastropods

Several gastropods are known to produce LAAOs (Figure 4B). The sea hare *Aplysia californica,* synthesizes a LAAO named escapin that is present in a separate gland from its substrates (Lys and Arg). The mixing of the secretion of both glands at the time of the attack of the predator generates a defensive mechanism against them [57]. Escapin shows antimicrobial activity against a range of bacteria, even when it is recombinantly expressed [58]. It has been shown that the antimicrobial activity of escapin depends not only on the hydrogen peroxide generated, but also on chemical compounds generated during the oxidation of l-lysine [59,60]. Another sea hare (*Aplysia punctata*) synthesizes a very similar LAAO (93% identical to escapin) whose antitumoral activity has been characterized [61]. Achacin is a LAAO isolated and characterized as an antimicrobial protein from the skin mucus of the giant snail *Achatina fulica* [62]. The cytotoxicity of achacin is mediated by two different mechanisms: hydrogen peroxide generation and apoptosis induction mediated by l-amino acids depletion [63].

### 2.5. Fungal LAAOs

Different flavoproteins with l-amino acid oxidase activities have been described in fungi. The *Neurospora crassa* and *Aspergillus nidulans* enzymes do not cluster in the same branch as the others, in spite of being close to them in the phylogenetic tree (Figure 4C). It is proposed that the utilization of amino acids as nitrogen source is their major physiological function. This role is also assumed for LAAOs in other eukaryotic microorganisms such as the single-cell alga *Chlamydomonas reinhardtii* [64,65]. Accordingly, fungal LAAOs usually have a broad substrate range to be able to use distinct amino acids for growing. This is the case of the LAAO from *Aspergillus nidulans* and *Neurospora crassa*, which are induced under nitrogen-limiting conditions [66,67]. Similarly, LAAO synthesized by *Laccaria bicolor* and *Hebeloma* sp. are also involved in amino acid catabolism and over-expressed under limiting concentrations of nitrogen. In addition, these enzymes seem to perform an important ecological role since they catalyze nitrogen mineralization from amino acids [68].

*Aspergillus fumigatus* produces another LAAO of wide substrate specificity, although it shows a certain degree of substrate preference. The enzyme is more specific for hydrophobic aromatic amino acids, namely l-tyrosine and l-phenylalanine [69]. *A. fumigatus* LAAO can be applied in the resolution of some racemic dl-amino acids, yielding optically pure d-amino acids [70].

Not all LAAOs reported in fungi are of broad substrate range and play a metabolic function. The l-lysine α-oxidase from *Trichoderma viride* (LysOX) exhibits high substrate specificity for l-lysine [71]. Recently, the crystal structure of LysOX has revealed similar overall structure to that of snake venom LAAOs. However, some residues in the funnel to the active site as well as the residues involved in the substrate side-chain recognition are distinct [72]. LysOX is expected to be a potential anti-cancer agent since it inhibits growth of cancer cells but shows relatively low cytotoxicity for normal cells [72,73]. Furthermore, LysOX is stable over a wide range of pH and temperature, so it is an attractive candidate for an enzyme-based l-lysine sensor [74,75]. More recently, an extracellular LAAO with antimicrobial activity has been isolated from *Trichoderma harzianum* ETS 323. This oxidase shows the best substrate specificity constant for l-phenylalanine, although it is also active on l-Lys, l-Glu and l-Ala. It is proposed that membrane permeabilization and reactive oxidative species production are involved in the mechanism responsible for its antibacterial activity [76]. Notably, LysOX and *T. harzianum* LAAO may be related with the capability of different *Trichoderma* species as biocontrol agents.

In addition, other fungal LAAOs with substrate specificity have been described. For instance, l-lysine oxidase from induced *Saccharomyces cerevisiae* has been reported as potential biosensor for l-lysine [77]. *Coprinus* sp. SF-1 LAAO catalyzes specifically the oxidative deamination of l-tryptophan, although it can also catalyze its decarboxylation depending on conditions. This oxidase is supposed to be involved in the l-tryptophan metabolism of the fungus [78].

### 2.6. Other Bacterial LAAOs

There are many bacterial enzymes showing LAAO activity that do not form a defined clustered or cannot be included in any of the previously described groups. In many cases, bacterial enzymes are more similar to proteins from eukaryotes than to other bacterial LAOs. An example would be glutamate oxidases from actinobacteria which are closely relate to LAAOs from animals. This observation suggests that these enzymes have an old evolutionary origin and that they have evolved to meet different physiological functions in distinct groups. In this section, we will discuss separately the most relevant features of the enzymes selected.

The flavoprotein glycine oxidase (GO, EC 1.4.3.19) catalyzes the oxidative deamination of some primary and secondary small-sized amines and d-amino acids to yield the corresponding α-keto acid, ammonium and hydrogen peroxide [79,80]. Glycine oxidase in *Bacillus subtilis* (ThiO), encoded by the *yjbR* gene, is involved in the biosynthesis of the thiazole moiety of thiamine [5]. In addition, another glycine oxidase has been described in the marine thermophilic bacterium *Geobacillus kaustophilus* (GoxK) [81]. This protein shares a similar substrate range and structure with the glycine oxidase from *B. subtilis*. Regarding applications, glycine oxidase can be used as a biosensor for glycine in biological samples [82], and in the degradation of the herbicide glyphosate [83,84]. Flavoproteins with sarcosine oxidase activity (SOX, EC 1.5.3.1), like the one from *Bacillus* sp. B-0618 (MSOX), exhibit substrate similarity with glycine oxidases since it catalyzes the oxidative demethylation of sarcosine (*N*-methylglycine) and other secondary amino acids (*N*-methyl-l-alanine, *N*-ethylglycine, and l-proline) [85]. However, MSOX does not cluster in the same group with glycine oxidases (Figure 2).

The hydrogen peroxide generated by a LAAO activity was described as a factor responsible for the competition of the oral *Streptococcus oligofermentans*, involved in the initial steps of dental biofilm formation, against *Streptococcus mutans*, one of the main caries-causing pathogens. The gene responsible for this action was cloned and its activity characterized as being held on 7 different amino acids, l-aspartic acid being the one with a higher rate of hydrogen peroxide production [21]. However, latter studies revealed that this flavoprotein is an aminoacetone oxidase (Aao) with a role in the antioxidant defence of *S. oligofermentans* against cellular ROS generation [22]. Phylogenetic analyses of several *Streptococcus* strains revealed that the gene in *S. oligofermentans* was acquired by horizontal gene transfer from a source closely related to *Streptococcus sanguinis* as a mechanism of adaptation to the aerobic conditions in the oral cavity [86]. In fact, BLAST analysis of *S. oligofermentans aao* gene product reveals that the most similar proteins are annotated as hypothetical or as pyridine nucleotide-disulfide oxidoreductases, such as in the case of *S. sanguinis*. In agreement with that, structure-function relationships of SoAAO set apart this protein from the oxidase/dehydrogenase class of flavoproteins [87].These enzymes have been investigated for their role in diverse processes such as epoxyalkanes degradation in *Xanthobacter* [88], as part of a redox system based on coenzyme A in *Staphylococcus aureus* [89] or as catalyzing the disulfide bond formation in *Chromobacterium violaceun* FK228 anticancer depsipeptide [90].

*Rhodococcus opacus* synthesizes a LAAO with broad substrate range that makes it interesting in some biotechnological applications such as the solving of racemic mixtures of amino acids [91]. The structure of the enzyme has been determined, what has contributed to a better understanding of its molecular mechanism of action [92]. Other LAAOs with broad substrate range have been cloned from different *Rhodococcus* strains [93]. As far as we know, no physiological function has been proposed for these enzymes, since they have been characterized in terms of their possible biotechnological applications. Other enzymes with broad substrate range were described in *Bacillus carotarum* [94], *Cellulomonas* [95], *Morganella morganii* [96] and *Corynebacterium* [97]. An example of LAAO with high substrate specificity is the glutamate oxidase synthesized by *Streptomyces* [98,99]. The physiological function of this extracellular enzyme is unknown. From the biotechnological point of view it is used as part of biosensors for the determination of glutamate [100].

Several LAAOs have been described in bacteria of the genus *Pseudomonas*. The strain *Pseudomonas* sp. AIU 813 synthesizes an enzyme with oxidase activity on l-Lys, ornithine and l-Arg [101]. This flavoprotein was lately reclassified as an l-amino acid/monooxygenase since the second activity was higher than the first [20]. Interestingly, the capacity to act as an oxidase or as a decarboxilating monooxygenase is shared by other enzymes such as the PAO (l-phenylalanine oxidase) from *Pseudomonas* P501 which is able to show different activities depending on the substrate [102,103]. *Pseudomonas savastanoi* TMO (tryptophan monooxygenase) does not show oxidase activity, but it can be revealed by mutagenesis [104].

VioA from *Chromobacterium violaceum* encodes an enzyme with l-tryptophan 2′,3′-oxidase activity that is involved in the synthesis of violacein [105]. An enzyme with the same enzymatic activity also participates in the synthesis of other secondary metabolites such as rebeccamycin and staurosporine produced by actimomycetes [106,107]. Interestingly the enzymes from the actinobacteria are not phylogenetically close to the one synthesized by *Chromobacterium* (Figure 2).

LAAOs with preference for basic amino acids have been described in cyanobacteria [108]. In *Synechococus* PCC 7942 that enzyme has been related to the use of Arg as nitrogen source [109].

## 3. Amino Acid Oxidases with Antimicrobial Activity in Marine Bacteria

The colonization of submerged surfaces is of great importance in marine environments. On the one hand, it can generate the biofouling of surfaces. On the other hand, bacteria associated with surfaces are important factors inducing the settlement and metamorphosis of larvae of higher organisms [110]. The genus *Pseudoalteromonas* has been recognized as an important component of the surface of many different marine surfaces [111,112]. *Marinomonas mediterranea* is a bacterium that forms part of the microbiota of the seagrass *Posidonia oceanica* [113]. From both, *Pseudoalteromonas* and *Marinomonas*, proteins with antibacterial activity were reported [114,115]. As described above, LodA is an amino acid oxidase with quinone cofactor. The autolytic protein of the marine bacterium *Pseudoalteromonas tunicata* (AlpP) also possesses lysine oxidase activity and high sequence similarity with LodA [13]. LodA and AlpP are involved in biofilm formation and mediate differentiation, dispersal, and phenotypic variation among the dispersed cells [13]. In two non-marine bacteria, *Caulobacter crescentus* and *Chromobacterium violaceum*, LodA-like proteins also generate hydrogen peroxide related with biofilm differentiation in those microorganisms, but the substrate of the activity was not reported [13].

Interestingly, other LodA-like proteins in the genus *Pseudoalteromonas* have been described as having antimicrobial activity. Two different strains of *Pseudoalteromonas flavipulchra* synthesize antimicrobial proteins of the Lod-A like group. *P. flavipulchra* JG1 produces an extracellular antibacterial protein (PfaP) with high similarity to LodA and AlpP [116]. The AAO of the strain C2 was reported to have a broad substrate range oxidizing l-Lys, l-Met, l-Glu, l-Leu, l-Gln, l-Tyr and l-Phe (Table S1.). The hydrogen peroxide generated by its catalysis mediates its antibacterial activity [117]. Another antibacterial LAAO with similar broad substrate range (l-Met, l-Gln, l-Leu, l-Phe, l-Glu, l-Trp, *etc.*) has been described in several *Pseudoalteromonas luteoviolacea* strains but the genes encoding those enzymes have not been reported yet [118]. As far as we know, the only case of LAAO activity not related to a quinoprotein in the genus *Pseudoalteromonas* has been describe in the strain B3, in which it has been associated to a flavoprotein with similarity to LASPOs [119,120] (Figure 3C). Genome mining of the genomes of *Pseudoalteromonas* strains, and other marine bacteria (data not shown) has revealed that they contain homologues to the gene encoding LASPO (*nadB)* as well as homologues to *nadA*. It remains to be determined whether these LASPOs participate in NAD synthesis or have evolved to meet an antimicrobial role. In the genomes of marine bacteria it is also possible to detect other genes encoding flavoproteins, some of them with similarity to amino oxidases. However, to the best of our knowledge, it has not been determined the actual enzymatic activity of the product of those genes or their possible antimicrobial activity.

The data available indicate that in the genomes of marine bacteria of the genus *Pseudoalteromonas* and *Marinomonas* it is common the presence of genes encoding proteins similar to LodA [12]. Although LodA-like proteins have been mainly described in marine bacteria, genes encoding proteins similar to them are present in approximately 1% of the microbial genomes sequenced, including microorganisms present in different environments. In the case of the freshwater *Rheinheimera aquatica* GR5, a protein with a peptide fragment with high similarity to LodA and AlpP was identified. This is an antimicrobial protein due to the hydrogen peroxide generation in the specific oxidation of l-Lys [121]. Nevertheless, it is important to bear in mind that in some microorganisms it is possible to detect several *lodA*-like genes [12]. Thus far, it has been shown that two of those genes in *Marinomonas* code for proteins with, respectively, l-lysine ε-oxidase and glycine oxidase activity. This observation suggests that they could have different, perhaps complementary physiological functions. LodA-like proteins may also constitute a reservoir of novel enzymatic activities of potential biotechnological interest.

## Supplementary Files

Supplementary File 1
