# Supplementary material for: Distribution in Different Organisms of Amino Acid Oxidases with FAD or a Quinone As Cofactor and Their Role as Antimicrobial Proteins in Marine Bacteria
Source: Mar Drugs. 2015 Dec 16;13(12):7403–18. doi: 10.3390/md13127073 (PMC4699246; doi:10.3390/md13127073)
Supplement: Supplementary File 1 [file marinedrugs-13-07073-s001.pdf]

Supplementary Materials: Distribution in Different Organisms of Amino Acid Oxidases with FAD or a Quinone As Cofactor and Their Role as Antimicrobial Proteins in Marine Bacteria

Jonatan C. Campillo-Brocal, Patricia Lucas-Elío and Antonio Sanchez-Amat *

Table S1. Amino acid oxidases (AAOs) from microbial sources. Marine microorganisms are shown in bold. * cofactor and/or activity attributed for high similarity with LodA. ND, not determined. NA, not accessible. LAAOs, L-amino acid oxidases. DAAOs, D-amino acid oxidases. LASPOs, L-aspartate oxidases. CTQ, cysteine tryptophilquinone.

Microorganism (Enzyme Name)	Activity (Main Substrate)	Cofactor	Oligomeric Structure/Mass Molecular	Various	Accession Number	Reference
			**AAOs with Quinone Cofactor (LodA-Like Proteins)**			
***Marinomonas mediterranea* MMB-1** (LodA)	L-Lysine ε-oxidase (L-Lys)	CTQ	Homotetramer (80.9 × 4 kDa). Crystal structure solved, PDB ID: 2YMW	Antimicrobial. Biofilms dispersion. Extracellular	AAY33849	[1,2]
***Marinomonas mediterranea* MMB-1** (GoxA)	Glycine oxidase (Gly)	CTQ	76.2 kDa	Other substrates: Gly ethyl ester	ADZ90918	[3,4]
***Pseudoalteromonas tunicata* D2** (AlpP)	L-Lysine ε-oxidase (L-Lys)	* CTQ	110 kDa	Antimicrobial. Biofilms dispersion	Q7X0I8	[5,6]
Chromobacterium violaceum	ND	ND	ND	Antimicrobial. Biofilms dispersion	AAQ60932	[6]
Caulobacter crescentus	ND	ND	ND	Antimicrobial. Biofilms dispersion	NP_419374	[6]
***Pseudoalteromonas flavipulchra* JG1** (PfaP)	* L-Lysine ε-oxidase (* L-Lys)	* CTQ	77 kDa	Antimicrobial. pI = 4.6	AFB71049	[7]
***Pseudoalteromonas flavipulchra* C2**	Broad spectrum oxidase (L-Lys > L-Met > L-Glu > L-Leu > L-Gln > L-Tyr > L-Phe)	ND	60 kDa	Antimicrobial. pI = 9.4. It contains a 9-residues peptide similar to AlpP	NA	[8]

Table S1. *Cont.*

Pseudoalteromonas luteoviolacea	Broad spectrum oxidase (L-Met > L-Gln > L-Leu > L-Phe > L-Glu > L-Trp)	ND	Oligomer (110 kDa)	Antimicrobial	NA	[9]
Rheinheimera aquatica GR5	* L-Lysine ε-oxidase (L-Lys)	* CTQ	Monomer (71 kDa)	Antimicrobial. pI = 3.6. It contains a 19-resiudes peptide similar to AlpP and LodA	NA	[10]
			**DAAOs**			
Rubrobacter xylanophilus (RxDAO)	D-Amino acid oxidase (neutral and basic D-aa)	FAD	Monomer (24,1 kDa)	Thermostable. pH opt = 7.5–10. Tª opt = 65 °C	BAP18969	[11]
Arthrobacter protophormiae (ApDAO)	D-Amino acid oxidase (neutral and hydrophobic D-aa)	FAD	Homodimer (34.6 × 2 kDa)	pI = 4.2. pH opt = 6.5–8.5	AAP70489	[12]
Rhodosporidium toruloides (RgDAO)	D-Amino acid oxidase	FAD	Homodimer (40 × 2 kDa). Crystal structure solved, PDB ID: 1C0L		P80324	[13]
Fusarium solani	D-Amino acid oxidase	FAD	40 kDa	Oxidizes Cephalosporin C	P24552	[14]
			**LASPOs**			
Bacillus subtilis 168	L-Aspartate oxidase (L-Asp)	FAD	Monomer-Dimer equilibrium (55–110 kDa)	NAD⁺ biosynthesis. Inhibited by iminoaspartate, succinate, fumarate, oxalacetate and D-Asp	NP_390665	[15]
Escherichia coli K12	L-Aspartate oxidase (L-Asp)	FAD	Monomer-Dimer equilibrium (60.3–120 kDa). Crystal structure solved, PDB ID: 1KNP	NAD⁺ biosynthesis. Inhibited by iminoaspartate, succinate and fumarate. pI = 5.6	P10902	[16,17]
***Pyrococcus horikoshii* OT-3**	L-Aspartate oxidase (L-Asp)	FAD	Homotrimer (51,925 × 3 kDa)	Thermostable. pH opt = 8.6. Tª opt = 90 °C	O57765	[18]
Sulfolobus tokodaii	L-Aspartate oxidase (L-Asp)	FAD	Monomer (53.6 kDa). Crystal structure solved, PDB ID 2E5V)	Other substrates: L-Asn. Tª opt = 79–87,5 °C	WP_0109792 15	[19,20]

Table S1. *Cont.*

***Pseudoalteromonas* sp. B3**	Broad spectrum oxidase (L-Leu > L-Lys > L-Tys > L-Asn > L-Gln > L-Met > L-cystine > L-Arg > L-Trp > L-Glu)	FAD	60 kDa	Homology with LASPOs	AJZ73816	[21]
**Fungal AAOs**						
Neurospora crassa	Broad spectrum oxidase (L-His > aminobutyric > L-canavanine > L-Tyr > D, L-Ornithine > D, L-Phe > L-Leu)	FAD	ND	L-aa catabolism	CAD21325	[22,23]
Aspergillus nidulans	Broad spectrum oxidase (L-His > aminobutyric > L-canavanine > L-Tyr > D, L-ornithine > D, L-Phe > L-Leu)	FAD	ND	L-aa catabolism	AAT84085	[24]
Laccaria bicolor S238N	Broad spectrum oxidase (L-Phe, L-His, L-Met, L-Leu, and L-Lys)	FAD	ND	L-aa catabolism. Nitrogen mineralization. pI = 6.2	DAA34975	[25,26]
Hebeloma cylindrosporum	Broad spectrum oxidase (L-Glu > L-Gln > L-Ornitina > L-Asn > L-Leu > L-His > L-Phe)	FAD	70 and 140 kDa	L-aa catabolism. Nitrogen mineralization. pI = 6.2. pH opt = 7–8	ADM80414	[25,26]
Aspergillus fumigatus P13	Broad spectrum oxidase (L-Tyr > L-Phe > L-Pro > L-Ser > L-Leu/L-Ala > L-Asp)	ND	ND	Non active on D-aa	ND	[27]
Trichoderma viride (LysOX)	L-Lysine α-oxidase (L-Lys)	FAD	Homodimer (55 × 2 kDa). Crystal structure solved, PDB ID: 3X0V	Antitumoral. Other substrates: L-Ornithine > L-Arg > L-Phe. pI = 4.2. pH opt = 4.5-10. Tª opt = 50 °C	BAR88116	[28,29]
Trichoderma harzianum ETS 323	Broad spectrum oxidase (L-Phe > L-Lys > L-Glu > L-Ala)	FAD	Monomer-Dimer equilibrium (63.5 kDa)	Biocontrol agent. Extracellular. pH opt = 7	ADD91592	[30,31]

Table S1. *Cont.*

Saccharomyces cerevisiae	L-Lysine oxidase (L-Lys)	ND	ND	L-Lys biosensor. Other substrates: L-Arg, L-Asn. pH opt = 7,5. Tª opt = 30 °C	NA	[32]
Coprinus sp. SF-1 (Tod)	L-Tryptophan oxidase (L-Trp and derivatives)	FAD	68 KDa	Other substrates: L -Phe and L-Tyr. Tª opt = 35–43 °C. pH opt = 7	NA	[33]
**Other enzymes with AAO activity**						
Bacillus subtilis 168 (GoxB)	Glycine oxidase (Gly)	FAD	Homotetramer (42 × 4 kDa). Crystal structure solved, PDB ID: 1NG4	Other substrates: Sarcosine, *N*-ethyl-Gly, D-Pro, D-Ala	O31616	[34,35]
Geobacillus *kaustophilus* HTA426 (GoxK)	Glycine oxidase (Gly)	FAD	Homotetramer (42 × 4 kDa)	Other substrates: Sarcosine, *N*-ethyl-Gly, D-Pro, D-Ala. Thermostable	BAD74908	[36]
Bacillus sp. B-0618 (MSox)	Sarcosine oxidase (sarcosine)	FAD	Monomer (43.8 kDa). Crystal structure solved, PDB ID: 2A89	Other substrates: *L*-Pro, *N*-ethyl-Gly, *N*-methyl- D L-Ala, *N*-methyl-D L-Val, *N*-methyl-L-Leu	BAA03967	[37,38]
Streptococcus oligofermentans (SoAAO)	Aminoacetone oxidase (aminoacteone)	FAD	43 kDa. Crystal structure solved, PDB ID: 4CNK	Antioxidant and microbial competition. Other substrates: L-Asp > L-Trp > L-Lys > L-Ile > L-Arg > L-Apn > L-Gln	ACA52024	[39–41]
Rhodococcus opacus DSM 43250	Broad spectrum oxidase (all the proteinogenic aa except Gly, L-Thr and L-Pro)	FAD	Homodimer (53 × 2 kDa). Crystal structure solved, PDB ID: 2JB2	pI = 4,8. pH opt = 8–9. Tª opt = 30 °C	AAL14831	[42,43]
Rhodococcus sp. AIU Z-35-1	Broad spectrum oxidase (L-Ala > Nα-Z-L-Lys > L-His > L-Tyr > L-ornithine > L-Gln)	FAD	Homodimer (51 × 2 kDa)	pI = 4.8. pH opt = 8–8,5. *N*-terminal homology with *Rhodococcus opacus* DSM 43250 LAO.	NA	[44,45]
Rhodococcus sp. AIU LAB-3	Broad spectrum oxidase (L-Ala > L-Gln > Nα-*Z*-L-Lys> L-rn > L-Arg > L-Phe > L-Met > L-Lys)	FAD	Homodimer (52,5×2 kDa)	pH opt = 6–8.5. Tª opt = 45 °C. *N*-terminal homology with *Rhodococcus opacus* DSM 43250 LAO.	NA	[45]

Table S1. *Cont.*

Bacillus carotarum 2Pfa	Broad spectrum oxidase (L-Leu > L-Lys > L-Arg > L-Met > L-Asn)	FAD	Homodimer (54×2 kDa)	Other substrates: D-isomers. pI = 4.8. pH opt = 8–8.5	NA	[46]
Cellulomonas cellulans AM8	Broad spectrum oxidase (all the proteinogenic aa except Gly, L-Pro and L-Thr)	FAD	55 kDa	Extracellular. pH opt=6,5-7,5	NA	[47]
Corynebacterium sp. A20	Broad spectrum oxidase (all the proteinogenic aa except L-Asp, L-Thr, L-Pro and Gly)	ND	130-140 kDa	L-aa catabolism	NA	[48]
Morganella morganii	Broad spectrum oxidase (L-Leu > L-Phe > L-Trp > L-Met > L-Tyr)	FAD	ND	Tª opt = 35–43 °C	NA	[49]
Streptomyces sp. X-119-6	L-Glutamate oxidase (L-Glu)	FAD	Hexamer $\alpha_2\beta_2\gamma_2$ [(42 + 17 + 10) × 2 kDa]. Crystal structure solved, PDB ID: 2E1M	L-Glu biosensor. Extracellular. pH opt = 7. Tª opt = 58 °C	BAB93449	[50–52]
Streptomyces endus	L-Glutamate oxidase (L-Glu)	FAD	Dimer (45 × 2 KDa)	pI = 6.2. pH opt = 6.5–8. Tª opt = 30–45 °C	NA	[53]
Streptomyces platensis NTU3304	L-Glutamate oxidase (L-Glu)	FAD	Heterotrimer of 78 kDa (39, 19 and 16 kDa)	L-Glu biosensor	AAK15071	[54]
Streptomyces violascens	L-Glutamate oxidase (L-Glu)	FAD	Monomer (60 kDa)	Other substrates: L-Gln	NA	[55]
Streptomyces sp. Z-11-6	L-Glutamate oxidase (L-Glu)	FAD	Tetramer $\alpha_2\beta_2$ [(25 + 22.5) × 2 kDa]	Extracellular	NA	[56]
Pseudomonas sp. AIU 813	L-Lysine α-oxidase/monooxygenase (L-Lys)	FAD	Homodimer (54.5 × 2 kDa). Crystal structure solved, PDB ID: 3WE0	Other substrates: L-Ornithine > L-Arg. Induced by L-Lys. pI = 4.6. pH opt = 7	BAO51829	[57,58]
Pseudomonas sp. P-501 (PAO)	L-Phenylalanine oxidase (deaminating and decarboxylating) (L-Phe)	FAD	Heterodimer $\alpha_2\beta_2$ [(10 + 60) × 2 kDa]. Crystal structure solved, PDB ID: 3AYJ	Other substrates: L-Tyr > L-Met > L-NorLeu > L-Trp. L-Phe is mainly oxygenated and L-Met is mainly oxidized	BAD66877	[59–61]
Pseudomonas savastanoi (TMO)	Tryptophan 2-monooxygenase (L-Trp)	FAD	62 kDa	Biosynthesis of indoleacetic acid	EFW87519	[62]

Table S1. *Cont.*

Ralstonia solanacearum (PTMO)	L-Tryptophan monooxygenase (L-Trp)	FAD	Heterodimer $\alpha_2\beta_2$ [(9.2 + 64.5) × 2 kDa].	Other substrates: L-Tyr, L-Trp, L-Met and L-Phe. L-Phe and L-Tyr are mainly oxygenated. L-Met is mainly oxidized. L-Trp is both oxygenated and oxidized. Homology with PAO	NA	[63]
Chromobacterium violacium (VioA)	L-Tryptophan oxidase (L-Trp and derivatives)	FAD	48 KDa	Violacein biosynthesis. pH opt = 9,25	Q9S3V1	[64]
Lechevalieria aerocolonigenes ATCC 39243 (RebO)	L-Tryptophan oxidase (L-Trp and derivatives)	FAD	Homodimer (54 × 2 kDa)	Rebeccamycin biosynthesis	BAC15750	[65]
Streptomyces sp. TP-A0274 (StaO)	L-Tryptophan oxidase (L-Trp and derivatives)	FAD	Homodimer (57x2 kDa)	Staurosporine biosynthesis. L-Trp biosensor. No activity on other L-aa. pH opt = 7–8	BAC55210	[66]
***Aquimarina* sp. antisso-27**	Broad spectrum oxidase (L-Leu > L-Ile > L-Met > L-Val)	ND	190 kDa	Algicide and antimicrobial. pI = 9,4	NA	[67]
Synechococcus elongatus PCC 6301 and PCC 7942	Basic L-aa oxidase (L-Arg > L-Lys > L-ornithine > L-His)	FAD	50 kDa	Catabolism of Arg. Periplasmic. pI= 8,5	CAA88452	[68,69]
Synechococcus cedrorum PCC 6908	Basic L-aa oxidase (L-Arg > L-Lys > L-ornithine > L-His)	FAD	Homodimer (49 × 2 kDa)	pI = 8.5	NA	[69]
Chlamydomonas reinhardtii	Broad spectrum oxidase (all the proteinogenic aa except L-Cys)	FAD	Oligomer $\alpha_x\beta_x$ [(66 + 135) × X kDa].	L-aa catabolism. Periplasmic	EDP07010	[70]

Table S2. Representative amino acid oxidases (AAOs) from animals. Marine organisms are shown in bold. ND, not determined.

Organism (Enzyme Name)	Activity (Main Substrate)	Cofactor	Oligomeric Structure /Mass Molecular	Various	Accession Number	Reference
			Gastropods			
Aplysia californica (Escapin)	L-Lysine and L-arginine oxidase (L-Lys, L-Arg)	FAD	Monomer (60 kDa)	Antimicrobial. Defence against predators	Q6IWZ0	[71]
Aplysia kurodai (Aplysianin A)	L-Lysine and L-arginine oxidase (L-Lys, L-Arg)	FAD	Homotetramer (85 × 4 kDa)	Antimicrobial	BAA11867	[72,73]
Aplysia californica (Aplysianin A)	L-Lysine and L-arginine oxidase (L-Lys, L-Arg)	FAD	Homotetramer (85 × 4 kDa)	Antimicrobial. It shares 85% amino acid sequence identity with Aplysianin A from *A. kurodai*	NP_001191524	[74]
Achatina fulica (Achacin)	Broad spectrum oxidase (L-Met > L-Leu > L-Trp > L-Lys > L-Arg > L-Phe > L-Cys > L-Asn)	FAD	56 kDa	Antimicrobial	CAA45871	[75,76]
			Vertebrates			
Sebastes schlegelii (SSAP)	L-Lysine oxidase (L-Lys)	FAD	Homodimer (53 × 2 kDa)	Antimicrobial. Innate immunity of fish skin	BAF43314	[77]
Danio rerio (Isoform X1)	ND	FAD	ND	Predicted LAAO	XP_009289996	NCBI
Bothrops jararacussu (BjsuLAAO)	Aromatic and hydrophobic amino acids oxidase (L-Met > L-Leu > L-Phe > L-Ile > L-Trp > L-Tyr)	FAD	Homodimer (56 × 2 kDa). Crystal structure solved, PDB ID: 4e0v	Antimicrobial. Present in the snake venom	AAR31182	[78]

Table S2. *Cont.*

Crotalus durissus cumanensis (CdcLAAO)	Aromatic and hydrophobic amino acids oxidase	FAD	Monomer (55 kDa)	Antimicrobial. Present in the snake venom	K9N7B7	[79]
Homo sapiens (IL4I1)	Aromatic amino acid oxidase (L-Phe > L-Trp > L-Tyr)	FAD	~70 kDa	Regulator of immune system	Q96RQ9	[80,81]
Homo sapiens (hDAAO)	D-Amino acid oxidase (D-Ala, D-Ser, D-Pro, Gly)	FAD	Homodimer (39.4 × 2 kDa)	Involved in D-Ser catabolism	NP_001908	[82]
Homo sapiens (DDO)	D-Aspartate oxidase (D-Asp, *N*-methyl-D-aspartate, D-Glu)	FAD	37 kDa	Involved in D-Asp catabolism	BAI44653	[83,84]

References

1. Lucas-Elio, P.; Gomez, D.; Solano, F.; Sanchez-Amat, A. The antimicrobial activity of marinocine, synthesized by *Marinomonas mediterranea*, is due to hydrogen peroxide generated by its lysine oxidase activity. *J. Bacteriol.* **2006**, *188*, 2493–2501.
2. Okazaki, S.; Nakano, S.; Matsui, D.; Akaji, S.; Inagaki, K.; Asano, Y. X-ray crystallographic evidence for the presence of the cysteine tryptophylquinone cofactor in L-lysine epsilon-oxidase from *Marinomonas mediterranea*. *J. Biochem.* **2013**, *154*, 233–236.
3. Campillo-Brocal, J.C.; Lucas-Elio, P.; Sanchez-Amat, A. Identification in *Marinomonas mediterranea* of a novel quinoprotein with glycine oxidase activity. *Microbiologyopen* **2013**, *2*, 684–694.
4. Chacon-Verdu, M.D.; Campillo-Brocal, J.C.; Lucas-Elio, P.; Davidson, V.L.; Sanchez-Amat, A. Characterization of recombinant biosynthetic precursors of the cysteine tryptophylquinone cofactors of *L*-lysine epsilon-oxidase and glycine oxidase from *Marinomonas mediterranea*. *Biochim. Biophys. Acta* **2015**, *1854*, 1123–1131.
5. James, S.G.; Holmstrom, C.; Kjelleberg, S. Purification and characterization of a novel antibacterial protein from the marine bacterium D2461. *Appl. Environ. Microbiol.* **1996**, *62*, 2783–2788.
6. Mai-Prochnow, A.; Lucas-Elio, P.; Egan, S.; Thomas, T.; Webb, J.S.; Sanchez-Amat, A.; Kjelleberg, S. Hydrogen peroxide linked to lysine oxidase activity facilitates biofilm differentiation and dispersal in several Gram-negative bacteria. *J. Bacteriol.* **2008**, *190*, 5493–5501.
7. Yu, M.; Wang, J.; Tang, K.; Shi, X.; Wang, S.; Zhu, W.M.; Zhang, X.H. Purification and characterization of antibacterial compounds of *Pseudoalteromonas flavipulchra* JG1. *Microbiology* **2012**, *158*, 835–842.
8. Chen, W.M.; Lin, C.Y.; Chen, C.A.; Wang, J.T.; Sheu, S.Y. Involvement of an L-amino acid oxidase in the activity of the marine bacterium *Pseudoalteromonas flavipulchra* against methicillin-resistant *Staphylococcus aureus*. *Enzyme Microb. Tech.* **2010**, *47*, 52–58.
9. Gomez, D.; Espinosa, E.; Bertazzo, M.; Lucas-Elio, P.; Solano, F.; Sanchez-Amat, A. The macromolecule with antimicrobial activity synthesized by *Pseudoalteromonas luteoviolacea* strains is an L-amino acid oxidase. *Appl. Microbiol. Biotechnol.* **2008**, *79*, 925–930.
10. Chen, W.M.; Lin, C.Y.; Sheu, S.Y. Investigating antimicrobial activity in *Rheinheimera* sp. due to hydrogen peroxide generated by L-lysine oxidase activity. *Enzyme Microb. Tech.* **2010**, *46*, 487–493.
11. Takahashi, S.; Furukawara, M.; Omae, K.; Tadokoro, N.; Saito, Y.; Abe, K.; Kera, Y. A highly stable D-amino acid oxidase of the thermophilic bacterium *Rubrobacter xylanophilus*. *Appl. Environ. Microbiol.* **2014**, *80*, 7219–7229.
12. Geueke, B.; Weckbecker, A.; Hummel, W. Overproduction and characterization of a recombinant D-amino acid oxidase from *Arthrobacter protophormiae*. *Appl. Microbiol. Biotechnol.* **2007**, *74*, 1240–1247.
13. Umhau, S.; Pollegioni, L.; Molla, G.; Diederichs, K.; Welte, W.; Pilone, M.S.; Ghisla, S. The X-ray structure of D-amino acid oxidase at very high resolution identifies the chemical mechanism of flavin-dependent substrate dehydrogenation. *Proc. Natl. Acad. Sci. USA* **2000**, *97*, 12463–12468.
14. Isogai, T.; Ono, H.; Ishitani, Y.; Kojo, H.; Ueda, Y.; Kohsaka, M. Structure and expression of cDNA for D-amino acid oxidase active against cephalosporin C from *Fusarium solani*. *J. Biochem.* **1990**, *108*, 1063–1069.
15. Marinoni, I.; Nonnis, S.; Monteferrante, C.; Heathcote, P.; Hartig, E.; Bottger, L.H.; Trautwein, A.X.; Negri, A.; Albertini, A.M.; Tedeschi, G. Characterization of L-aspartate oxidase and quinolinate synthase from *Bacillus subtilis*. *FEBS J.* **2008**, *275*, 5090–5107.
16. Bossi, R.T.; Negri, A.; Tedeschi, G.; Mattevi, A. Structure of FAD-bound L-aspartate oxidase: insight into substrate specificity and catalysis. *Biochemistry* **2002**, *41*, 3018–3024.
17. Seifert, J.; Kunz, N.; Flachmann, R.; Laufer, A.; Jany, K.D.; Gassen, H.G. Expression of the *E. coli nadB* gene and characterization of the gene product L-aspartate oxidase. *Biol. Chem. Hoppe Seyler* **1990**, *371*, 239–248.
18. Sakuraba, H.; Satomura, T.; Kawakami, R.; Yamamoto, S.; Kawarabayasi, Y.; Kikuchi, H.; Ohshima, T. L-aspartate oxidase is present in the anaerobic hyperthermophilic archaeon *Pyrococcus horikoshii* OT-3: Characteristics and role in the de novo biosynthesis of nicotinamide adenine dinucleotide proposed by genome sequencing. *Extremophiles* **2002**, *6*, 275–281.
19. Bifulco, D.; Pollegioni, L.; Tessaro, D.; Servi, S.; Molla, G. A thermostable L-aspartate oxidase: A new tool for biotechnological applications. *Appl. Microbiol. Biotechnol.* **2013**, *97*, 7285–7295.

20. Sakuraba, H.; Yoneda, K.; Asai, I.; Tsuge, H.; Katunuma, N.; Ohshima, T. Structure of L-aspartate oxidase from the hyperthermophilic archaeon *Sulfolobus tokodaii*. *Biochim. Biophys. Acta* **2008**, *1784*, 563–571.
21. Yu, Z.; Zhou, N.; Qiao, H.; Qiu, J. Identification, cloning, and expression of L-amino acid oxidase from marine *Pseudoalteromonas* sp. B3. *Sci. World J.* **2014**, *2014*, 979858, doi:10.1155/2014/979858.
22. Niedermann, D.M.; Lerch, K. Molecular cloning of the L-amino-acid oxidase gene from *Neurospora crassa*. *J. Biol. Chem.* **1990**, *265*, 17246–17251.
23. Thayer, P.S.; Horowitz, N.H. The L-amino acid oxidase of *Neurospora*. *J. Biol. Chem.* **1951**, *192*, 755–767.
24. Davis, M.A.; Askin, M.C.; Hynes, M.J. Amino acid catabolism by an *areA*-regulated gene encoding an L-amino acid oxidase with broad substrate specificity in *Aspergillus nidulans*. *Appl. Environ. Microbiol.* **2005**, *71*, 3551–3555.
25. Nuutinen, J.T.; Timoneni, S. Identification of nitrogen mineralization enzymes, L-amino acid oxidases, from the ectomycorrhizal fungi *Hebeloma* spp. and *Laccaria bicolor*. *Mycol. Res.* **2008**, *112*, 1453–1464.
26. Nuutinen, J.T.; Marttinen, E.; Soliymani, R.; Hilden, K.; Timonen, S. L-Amino acid oxidase of the fungus *Hebeloma cylindrosporum* displays substrate preference towards glutamate. *Microbiology* **2012**, *158*, 272–283.
27. Singh, S.; Gogoi, B.K.; Bezbaruah, R.L. Optimization of medium and cultivation conditions for L-amino acid oxidase production by *Aspergillus fumigatus*. *Can. J. Microbiol.* **2009**, *55*, 1096–1102.
28. Kusakabe, H.; Kodama, K.; Kuninaka, A.; Yoshino, H.; Misono, H.; Soda, K. A new antitumor enzyme, L-lysine alpha-oxidase from *Trichoderma viride*. Purification and enzymological properties. *J. Biol. Chem.* **1980**, *255*, 976–981.
29. Amano, M.; Mizuguchi, H.; Sano, T.; Kondo, H.; Shinyashiki, K.; Inagaki, J.; Tamura, T.; Kawaguchi, T.; Kusakabe, H.; Imada, K.; *et al*. Recombinant expression, molecular characterization and crystal structure of antitumor enzyme, L-lysine-a-oxidase from *Trichoderma viride*. *J. Biochem.* **2015**, *157*, 549–559.
30. Yang, C.A.; Cheng, C.H.; Lo, C.T.; Liu, S.Y.; Lee, J.W.; Peng, K.C. A novel L-amino acid oxidase from *Trichoderma harzianum* ETS 323 associated with antagonism of *Rhizoctonia solani*. *J. Agric. Food Chem.* **2011**, *59*, 4519–4526.
31. Yang, C.A.; Cheng, C.H.; Liu, S.Y.; Lo, C.T.; Lee, J.W.; Peng, K.C. Identification of antibacterial mechanism of L-amino acid oxidase derived from *Trichoderma harzianum* ETS 323. *FEBS J.* **2011**, *278*, 3381–3394.
32. Akyilmaz, E.; Erdogan, A.; Ozturk, R.; Yasa, I. Sensitive determination of L-lysine with a new amperometric microbial biosensor based on *Saccharomyces cerevisiae* yeast cells. *Biosens. Bioelectron.* **2007**, *22*, 1055–1060.
33. Furuya, Y.; Sawada, H.; Hirahara, T.; Ito, K.; Ohshiro, T.; Izumi, Y. A novel enzyme, L-tryptophan oxidase, from a basidiomycete, *Coprinus* sp. SF-1: Purification and characterization. *Biosci. Biotechnol. Biochem.* **2000**, *64*, 1486–1493.
34. Nishiya, Y.; Imanaka, T. Purification and characterization of a novel glycine oxidase from *Bacillus subtilis*. *FEBS Lett.* **1998**, *438*, 263–266.
35. Settembre, E.C.; Dorrestein, P.C.; Park, J.H.; Augustine, A.M.; Begley, T.P.; Ealick, S.E. Structural and mechanistic studies on ThiO, a glycine oxidase essential for thiamin biosynthesis in *Bacillus subtilis*. *Biochemistry* **2003**, *42*, 2971–2981.
36. Martinez-Martinez, I.; Navarro-Fernandez, J.; Garcia-Carmona, F.; Takami, H.; Sanchez-Ferrer, A. Characterization and structural modeling of a novel thermostable glycine oxidase from *Geobacillus kaustophilus* HTA426. *Proteins* **2008**, *70*, 1429–1441.
37. Suzuki, H. Sarcosine oxidase: structure, function, and the application to creatinine determination. *Amino Acids* **1994**, *7*, 27–43.
38. Trickey, P.; Wagner, M.A.; Jorns, M.S.; Mathews, F.S. Monomeric sarcosine oxidase: structure of a covalently flavinylated amine oxidizing enzyme. *Structure* **1999**, *7*, 331–345.
39. Molla, G.; Nardini, M.; Motta, P.; D'Arrigo, P.; Panzeri, W.; Pollegioni, L. Aminoacetone oxidase from *Streptococcus oligofermentas* belongs to a new three-domain family of bacterial flavoprotein. *Biochem. J.* **2014**, *464*, 387–399.

40. Tong, H.; Chen, W.; Shi, W.; Qi, F.; Dong, X. SO-LAAO, a novel L-amino acid oxidase that enables *Streptococcus oligofermentans* to over-compete *Streptococcus* mutans by generating H_2O_2 from peptone. *J. Bacteriol.* **2008**, *190*, 4716–4721.
41. Zhou, P.; Liu, L.; Tong, H.; Dong, X. Role of operon *aaoSo-mutT* in antioxidant defense in *Streptococcus oligofermentans*. *PLoS ONE* **2012**, *7*, e38133, doi:10.1371/journal.pone.0038133.
42. Faust, A.; Geueke, B.; Niefind, K.; Hummel, W.; Schomburg, D. Crystallization and preliminary X-ray analysis of a bacterial L-amino-acid oxidase from *Rhodococcus opacus*. *Acta Crystallogr. Sect. F. Struct. Biol. Cryst. Commun.* **2006**, *62*, 279–281.
43. Geueke, B.; Hummel, W. Heterologous expression of *Rhodococcus opacus* L-amino acid oxidase in *Streptomyces lividans*. *Protein Expr. Purif.* **2003**, *28*, 303–309.
44. Isobe, K.; Nagasawa, S. Characterization of Nalpha-benzyloxycarbonyl-L-lysine oxidizing enzyme from *Rhodococcus* sp. AIU Z-35–1. *J. Biosci. Bioeng.* **2007**, *104*, 218–223.
45. Isobe, K.; Satou, S.; Matsumoto, E.; Yoshida, S.; Yamada, M.; Hibi, M.; Ogawa, J. Characterization and application of a L-specific amino acid oxidase from *Rhodococcus* sp. AIU LAB-3. *J. Biosci. Bioeng.* **2013**, *115*, 613–617.
46. Brearley, G.M.; Price, C.P.; Atkinson, T.; Hammond, P.M. Purification and partial characterisation of a broad-range L-amino acid oxidase from *Bacillus carotarum* 2Pfa isolated from soil. *Appl. Microbiol. Biotechnol.* **1994**, *41*, 670–676.
47. Braun, M.; Kim, J.M.; Schmid, R.D. Purification and some properties of an extracellular L-amino acid oxidase from *Cellulomonas cellulans* AM8 isolated from soil. *Appl. Microbiol. Biotechnol.* **1992**, *37*, 594–598.
48. Coudert, M. Charcterization and physiological function of a soluble L-amino acid oxidase in *Corynebacterium*. *Arch. Microbiol.* **1975**, *102*, 151–153.
49. Bouvrette, P.; Luong, J.H.T. Purification and further characterization of an L-phenylalanine oxidase from *Morganella morganii*. *Appl. Biochem. Biotechnol.* **1994**, *48*, 61–74.
50. Arima, J.; Tamura, T.; Kusakabe, H.; Ashiuchi, M.; Yagi, T.; Tanaka, H.; Inagaki, K. Recombinant expression, biochemical characterization and stabilization through proteolysis of an L-glutamate oxidase from *Streptomyces* sp. X-119–6. *J. Biochem.* **2003**, *134*, 805–812.
51. Utsumi, T.; Arima, J.; Sakaguchi, C.; Tamura, T.; Sasaki, C.; Kusakabe, H.; Sugio, S.; Inagaki, K. Arg305 of *Streptomyces* L-glutamate oxidase plays a crucial role for substrate recognition. *Biochem. Biophys. Res. Commun.* **2012**, *417*, 951–955.
52. Kusakabe, H.; Midorikawa, Y.; Fujishima, T.; Kuninaka, A.; Yoshino, H. Purification and properties of a new enzyme, L-glutamate oxidase, from *Streptomyces* sp. X-119–6 grown on wheat bran. *Agric. Biol. Chem.* **1983**, *47*, 1323–1328.
53. Bohmer, A.; Muller, A.; Passarge, M.; Liebs, P.; Honeck, H.; Muller, H.G. A novel L-glutamate oxidase from *Streptomyces endus*. Purification and properties. *Eur. J. Biochem.* **1989**, *182*, 327–332.
54. Chen, C.Y.; Wu, W.T.; Huang, C.J.; Lin, M.H.; Chang, C.K.; Huang, H.J.; Liao, J.M.; Chen, L.Y.; Liu, Y.T. A common precursor for the three subunits of L-glutamate oxidase encoded by *gox* gene from *Streptomyces platensis* NTU3304. *Can. J. Microbiol.* **2001**, *47*, 269–275.
55. Kamei, T.; Asano, K.; Suzuki, H.; Matsuzaki, M.; Nakamura, S. L-Glutamate oxidase from *Streptomyces violascens*. I. Production, isolation and some properties. *Chem. Pharm. Bull.* **1983**, *31*, 1307–1314.
56. Sukhacheva, M.V.; Zhuravleva, N.I. Properties and prospects of practical use of extracellular L-glutamate oxidase from *Streptomyces* sp. Z-11–6. *Prikl. Biokhim. Mikrobiol.* **2004**, *40*, 173–177.
57. Isobe, K.; Asami, S.; Domon, H.; Fukuta, Y.; Asano, Y. Purification and characterization of an L-amino acid oxidase from *Pseudomonas* sp. AIU 813. *J. Biosci. Bioeng.* **2012**, *114*, 257–261.
58. Matsui, D.; Im, D.H.; Sugawara, A.; Fukuta, Y.; Fushinobu, S.; Isobe, K.; Asano, Y. Mutational and crystallographic analysis of L-amino acid oxidase/monooxygenase from *Pseudomonas* sp. AIU 813: Interconversion between oxidase and monooxygenase activities. *FEBS Open Bio.* **2014**, *4*, 220–228.
59. Ida, K.; Kurabayashi, M.; Suguro, M.; Hiruma, Y.; Yamamoto, M.; Suzuki, H. Structural basis of proteolytic activation of L-phenylalanine oxidase from *Pseudomonas* sp. P-501. *J. Biol. Chem.* **2008**, *24*, 16584–16590.
60. Ida, K.; Suguro, M.; Suzuki, H. High resolution x-ray crystal structures of L-phenylalanine oxidase (deaminating and decarboxylating) from *Pseudomonas* sp. P-501. Structures of the enzyme-ligand complex and catalytic mechanism. *J. Biochem.* **2011**, *150*, 659–669.

61. Koyama, H. Oxidation and oxygenation of L-amino acids catalyzed by a L-phenylalanine oxidase (deaminating and decarboxylating) from *Pseudomonas* sp. P-501. *J. Biochem.* **1984**, *96*, 421–427.
62. Sobrado, P.; Fitzpatrick, P.F. Analysis of the role of the active site residue Arg98 in the flavoprotein tryptophan 2-monooxygenase, a member of the L-amino oxidase family. *Biochemistry* **2003**, *42*, 13826–13832.
63. Kurosawa, N.; Hirata, T.; Suzuki, H. Characterization of putative tryptophan monooxygenase from *Ralstonia solanasearum*. *J. Biochem.* **2009**, *146*, 23–32.
64. Balibar, C.J.; Walsh, C.T. *In vitro* biosynthesis of violacein from L-tryptophan by the enzymes VioA-E from *Chromobacterium violaceum*. *Biochemistry* **2006**, *45*, 15444–15457.
65. Nishizawa, T.; Aldrich, C.; Sherman, D.H. Molecular analysis of the rebeccamycin L-amino acid oxidase from *Lechevalieria aerocolonigenes* ATCC 39243. *J. Bacteriol.* **2005**, *187*, 2084–2092.
66. Kameya, M.; Onaka, H.; Asano, Y. Selective tryptophan determination using tryptophan oxidases involved in bis-indole antibiotic biosynthesis. *Anal. Biochem.* **2013**, *438*, 124–132.
67. Chen, W.M.; Sheu, F.S.; Sheu, S.Y. Novel L-aminoacid oxidase with algicidal activity against toxic cyanobacterium *Microcystis aeruginosa* synthesized by a bacterium *Aquimarina* sp. *Enzyme Microb. Technol.* **2011**, *49*, 372–379.
68. Pistorius, E.K.; Voss, H. Presence of an amino acid oxidase in photosystem II of *Anacystis nidulans*. *Eur. J. Biochem.* **1982**, *126*, 203–209.
69. Gau, A.E.; Heindl, A.; Nodop, A.; Kahmann, U.; Pistorius, E.K. L-Amino acid oxidases with specificity for basic L-amino acids in cyanobacteria. *Z. Naturforsch. C Biosci.* **2007**, *62*, 273–284.
70. Vallon, O.; Bulte, L.; Kuras, R.; Olive, J.; Wollman, F.A. Extensive accumulation of an extracellular L-amino-acid oxidase during gametogenesis of *Chlamydomonas reinhardtii*. *Eur. J. Biochem.* **1993**, *215*, 351–360.
71. Yang, H.; Johnson, P.M.; Ko, K.C.; Kamio, M.; Germann, M.W.; Derby, C.D.; Tai, P.C. Cloning, characterization and expression of escapin, a broadly antimicrobial FAD-containing L-amino acid oxidase from ink of the sea hare *Aplysia californica*. *J. Exp. Biol.* **2005**, *208*, 3609–3622.
72. Jimbo, M.; Nakanishi, F.; Ryuichi Sakai, F.; Koji Muramoto, F.; Hisao Kamiya, F. Characterization of L-amino acid oxidase and antimicrobial activity of aplysianin A, a sea hare-derived antitumor-antimicrobial protein. *Fisheries Sci.* **2003**, *69*, 1240–1246.
73. Kamiya, H.; Muramoto, K.; Yamazaki, M. Aplysianin-A, an antibacterial and antineoplastic glycoprotein in the albumen gland of a sea hare, *Aplysia kurodai*. *Experientia* **1986**, *42*, 1065–1067.
74. Cummins, S.F.; Nichols, A.E.; Amare, A.; Hummon, A.B.; Sweedler, J.V.; Nagle, G.T. Characterization of *Aplysia* enticin and temptin, two novel water-borne protein pheromones that act in concert with attractin to stimulate mate attraction. *J. Biol. Chem.* **2004**, *279*, 25614–25622.
75. Ehara, T.; Kitajima, S.; Kanzawa, N.; Tamiya, T.; Tsuchiya, T. Antimicrobial action of achacin is mediated by L-amino acid oxidase activity. *FEBS Lett.* **2002**, *531*, 509–512.
76. Obara, K.; Otsuka-Fuchino, H.; Sattayasai, N.; Nonomura, Y.; Tsuchiya, T.; Tamiya, T. Molecular cloning of the antibacterial protein of the giant African snail, *Achatina fulica* Ferussac. *Eur. J. Biochem.* **1992**, *209*, 1–6.
77. Kitani, Y.; Tsukamoto, C.; Zhang, G.; Nagai, H.; Ishida, M.; Ishizaki, S.; Shimakura, K.; Shiomi, K.; Nagashima, Y. Identification of an antibacterial protein as L-amino acid oxidase in the skin mucus of rockfish *Sebastes schlegeli*. *FEBS J.* **2007**, *274*, 125–136.
78. Ullah, A.; Souza, T.A.; Abrego, J.R. B.; Betzel, C.; Murakami, M.T.; Arni, R.K. Structural insights into selectivity and cofactor binding in snake venom L-amino acid oxidases. *Biochem. Biophys. Res. Commun.* **2012**, *421*, 124–128.
79. Vargas, L.J.; Quintana, J.C.; Pereanez, J.A.; Nunez, V.; Sanz, L.; Calvete, J. Cloning and characterization of an antibacterial L-amino acid oxidase from *Crotalus durissus cumanensis* venom. *Toxicon* **2013**, *64*, 1–11.
80. Boulland, M.L.; Marquet, J.; Molinier-Frenkel, V.; Moller, P.; Guiter, C.; Lasoudris, F.; Copie-Bergman, C.; Baia, M.; Gaulard, P.; Leroy, K.; Castellano, F. Human IL4I1 is a secreted L-phenylalanine oxidase expressed by mature dendritic cells that inhibits T-lymphocyte proliferation. *Blood* **2007**, *110*, 220–227.
81. Mason, J.M.; Naidu, M.D.; Barcia, M.; Porti, D.; Chavan, S.S.; Chu, C.C. IL-4-induced gene-1 is a leukocyte L-amino acid oxidase with an unusual acidic pH preference and lysosomal localization. *J. Immunol.* **2004**, *173*, 4561–4567.

82. Molla, G.; Sacchi, S.; Bernasconi, M.; Pilone, M.S.; Fukui, K.; Pollegioni, L. Characterization of human D-amino acid oxidase. *FEBS Lett.* **2006**, *580*, 2358–2364.
83. Katane, M.; Saitoh, Y.; Seida, Y.; Sekine, M.; Furuchi, T.; Homma, H. Comparative characterization of three D-aspartate oxidases and one D-amino acid oxidase from *Caenorhabditis elegans*. *Chem. Biodivers.* **2010**, *7*, 1424–1434.
84. Setoyama, C.; Miura, R. Structural and Functional Characterization of the Human Brain D-aspartate oxidase. *J. Biochem.* **1997**, *121*, 798–803.
